# Implementing Kanyini GAP, a pragmatic randomised controlled trial in Australia: findings from a qualitative study

**DOI:** 10.1186/s13063-015-0956-y

**Published:** 2015-09-23

**Authors:** Hueiming Liu, Luciana Massi, Anne-Marie Eades, Kirsten Howard, David Peiris, Julie Redfern, Tim Usherwood, Alan Cass, Anushka Patel, Stephen Jan, Tracey-Lea Laba

**Affiliations:** The George Institute for Global Health, University of Sydney, PO Box M201, Missenden Road, Camperdown, NSW 2050 Australia; Faculty of Pharmacy, University of Sydney, Sydney, Australia; Sydney School of Public Health, University of Sydney, Sydney, NSW 2006 Australia; Department of General Practice, Sydney Medical School Westmead, University of Sydney, Sydney, NSW 2006 Australia; Menzies School of Health Research, Charles Darwin University, Casuarina, NT 0811 Australia

**Keywords:** Pragmatic randomised controlled trial, Primary healthcare, Indigenous health services, Implementation, Clinical trial

## Abstract

**Background:**

Pragmatic randomised controlled trials (PRCTs) aim to assess intervention effectiveness by accounting for ‘real life’ implementation challenges in routine practice. The methodological challenges of PRCT implementation, particularly in primary care, are not well understood. The Kanyini Guidelines Adherence to Polypill study (Kanyini GAP) was a recent primary care PRCT involving multiple private general practices, Indigenous community controlled health services and private community pharmacies. Through the experiences of Kanyini GAP participants, and using data from study materials, this paper identifies the critical enablers and barriers to implementing a PRCT across diverse practice settings and makes recommendations for future PRCT implementation.

**Methods:**

Qualitative data from 94 semi-structured interviews (47 healthcare providers (pharmacists, general practitioners, Aboriginal health workers; 47 patients) conducted for the process evaluation of Kanyini GAP was used. Data coded to ‘trial impact’, ‘research motivation’ and ‘real world’ were explored and triangulated with data extracted from study materials (e.g. Emails, memoranda of understanding and financial statements).

**Results:**

PRCT implementation was facilitated by an extensive process of relationship building at the trial outset including building on existing relationships between core investigators and service providers. Health providers’ and participants’ altruism, increased professional satisfaction, collaboration, research capacity and opportunities for improved patient care enabled implementation. Inadequate research infrastructure, excessive administrative demands, insufficient numbers of adequately trained staff and the potential financial impact on private practice were considered implementation barriers. These were largely related to this being the first experience of trial involvement for many sites. The significant costs of addressing these barriers drew study resources from the task of achieving recruitment targets.

**Conclusions:**

Conducting PRCTs is crucial to generating credible evidence of intervention effectiveness in routine practice. PRCT implementation needs to account for the particular challenges of implementing collaborative research across diverse stakeholder organisations. Reliance on goodwill to participate is crucial at the outset. However, participation costs, particularly for organisations with little or no research experience, can be substantial and should be factored into PRCT funding models. Investment in a pool to fund infrastructure in the form of primary health research networks will offset some of these costs, enabling future studies to be implemented more cost-effectively.

**Trial registration:**

ACTRN126080005833347

**Electronic supplementary material:**

The online version of this article (doi:10.1186/s13063-015-0956-y) contains supplementary material, which is available to authorized users.

## Background

Randomised controlled trials are generally seen as the ‘gold standard’ for assessing the efficacy of health sector interventions. Challenges in applying the evidence based on explanatory trials of interventions tested in optimal conditions have led to a growing emphasis for pragmatic randomised controlled trials (PRCTs). PRCTs involve a comparison of interventions and using health outcome measures that are relevant to ‘real-world’ healthcare delivery. This allows for generalisability of the PRCTs’ findings which may be more accessible to decision-makers and thus be translated into practice and policy [1–6]. PRCT interventions are often multifaceted with multipurposed analyses and the provider and the recipient of an intervention may not only be the health professionals and patients respectively but can be other members in the health system [2].

Designing PRCTs is not straightforward. For instance, in primary care settings, given the broad spectrum of disease presentation and diverse practice settings, maximising generalisability in a PRCT without overly compromising reliability or accuracy has proven difficult [8]. In this regard, strategies to deal with design issues such as unblinded treatment allocation and recruiting representative participants have been suggested [9]. More recently, a tool of ten domains known as the Pragmatic-Explanatory Continuum Indicator Summary (PRECIS) was developed by Thorpe et al. as a guide for researchers in designing PRCTs [10, 11]. The ten domains include participant eligibility criteria, intervention and comparison flexibility and expertise, follow-up intensity, participant compliance and participant adherence to study protocol, selecting and analysing primary outcomes which are relevant to clinical practice [7]. It is thought that by capturing ‘real-life’ practice variation the evidence generated will be more relevant to policy-makers [7].

The Kanyini Guidelines Adherence with the Polypill study (Kanyini GAP) provides a recent example of a PRCT that was implemented within Australian primary care. Kanyini GAP sought to explore whether a strategy based on the use of a fixed-dose combination pill (polypill), comprising low-dose aspirin, a statin and two blood pressure lowering agents, would improve patient adherence to and provider prescribing of evidence-based cardiovascular disease (CVD) preventive medications [12, 13]. This trial was conducted in primary care rather than in hospitals reflecting the setting where, in practice, prevention and early management of cardiovascular disease is most likely to take place [14].

**Fig. 1:**
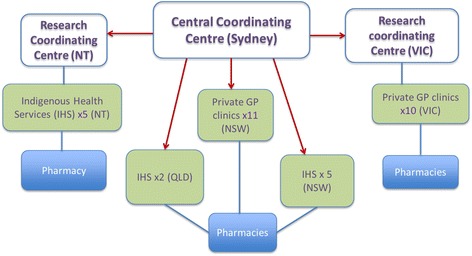
Organisational structure of the Kanyini GAP study. Kanyini GAP was conducted in 33 sites across urban, rural and remote Australia and recruited 623 patients through 12 Indigenous health services and 21 private general practices. There was one central coordinating centre based in Sydney and two regional coordinating centres based in Alice Springs and Victoria, which recruited and coordinated the sites in NSW, Queensland, Victoria and the Northern Territory. Each ‘site’ included either a general practice clinic or an Indigenous health service and 1–3 community pharmacies

Kanyini GAP included a range of diverse practice sizes and settings across Australia: 12 Indigenous Health Services (IHS) (which were 11 Aboriginal Community Controlled Health Services and 1 government-run health service) and 21 private mainstream general practices. Medications were dispensed through community pharmacies with patients in both treatment groups required to pay for their medicines at the prevailing co-payment rate. By incorporating these design features, the study sought to mimic the systems through which the comparative treatments would be delivered in practice; therefore, potentially yielding more generalisable assessments of ‘real-life’ effectiveness. Figure 1 shows the organisation of the trial management between the research coordinating centres and primary care services. A Consolidated Standards of Reporting Trials (CONSORT) flow diagram and checklist of this completed randomised controlled trial (RCT) are included as additional documents (see Additional files 1 and 2). Despite Kanyini GAP being designed according to recommendations in the PRCT-related literature, a number of problems were encountered in implementation: recruitment fell considerably short of expected targets (*n* = 623 c.f. 1000), challenges related to stakeholders not having prior research experience yet required to comply with Good Clinical Practice [15], study duration was considerably longer than expected and the costs exceeded the projected budget.

At present, there is very little published evidence that describes the experience of implementing a PRCT in primary care from the perspectives of the participants. To address this gap in evidence, this paper aims to identify the critical enablers and barriers to implementing Kanyini GAP. Drawing on the experiences of patients and providers participating in Kanyini GAP and key trial documentation, we sought to make recommendations for the future implementation of PRCTs in primary healthcare settings.

## Methods

This study uses qualitative data from the overall process evaluation of Kanyini GAP [16]. A predefined protocol for the process evaluation was used [16]. The methods are described across the three domains as specified in the consolidated criteria for reporting of qualitative studies developed by Tong et al. from a review of established guidelines and qualitative studies [17].

### Research team and reflexivity

Study investigators (TU, SJ, JR, TL, DP, and AC) who were involved in the design and implementation of Kanyini GAP developed the interview guides which were iteratively revised to explore themes and issues emerging from earlier interviews. Views about the polypill strategy in CVD management, patient satisfaction or problems with the polypill, issues regarding trial implementation, and perspectives of translation of the polypill into clinical practice were key domains of the interview guides. The interviews were conducted by a team of seven interviewers. Two of the interviewers had existing relationships with some of the participants interviewed as they were research coordinators in the trial but the other interviewers were not known to the participants prior to the interviews. The team had diverse backgrounds (health economics, nursing, pharmacy, Indigenous health), varied experience in qualitative research and three were Indigenous and four non-Indigenous researchers.

### Study design

Site recruitment for the Kanyini GAP trial was initiated with the general practitioners (GPs) of private practices and IHSs, as well as the board members of IHSs. A critical enabler to the recruitment of GPs was the extensive process of undertaking relationship building and leveraging existing networks amongst Kanyini GAP chief investigators. In particular, prior to Kanyini GAP, a relationship between the research-team and several of the IHSs existed as part of the Kanyini Vascular Collaboration – a chronic disease-based research collaboration between the two research institutes and participating IHSs around Australia.

Participants were purposively recruited for the qualitative study from these participating Kanyini GAP sites based on maximum variation of specified variables which were based on adherence literature which may affect participants’ experience with a polypill-based strategy and also variables which may impact trial implementation. We used the sampling matrix to select patients based on these characteristics; for patients these were location, age, gender, ethnicity, primary versus secondary CVD, and self-reported adherence at baseline; and for providers variables included location and profession [18]. All health providers and patients were sent an invitation letter outlining the study and its objectives, and followed-up by a phone call by the project coordinator (LM). Five patients declined to be interviewed and two patients were not available, and all health providers agreed to be interviewed. Written informed consent was obtained from all participants.

Most face-to-face interviews, which ranged from 30 min to an hour, were conducted either at home or at the health service, and audio-recorded. Two participants from interstate were not available for a face-to-face interview. Phone interviews were conducted with these two participants and audio-recorded. One researcher LM was involved in almost all the interviews to ensure consistency [18]. She conducted 40 with another interviewer and 42 alone. LM coordinated the study and was trained in qualitative methods. She conducted preliminary thematic data analysis alongside the interviews. Thematic saturation was achieved and interviews stopped.

### Analysis

Interviews were professionally transcribed. At the completion of all the interviews, two researchers (HL and LM) used NVivo 9 (QSR International, Melbourne, VIC, Australia) to code and manage the qualitative data. Using the constant comparative method [19], these researchers coded line by line the same twelve transcripts independently (six patients and six health providers across the variables) through three iterative stages and an initial coding framework was developed. Insights about the local setting, context of the interviews and empirical results of the PRCT were documented and used to aid interpretation and triangulation [20, 21]. The overarching coding framework (which is included as an Additional file 3) was developed for provider and patient interviews and refined with the study investigators and the interview team. This included two IHSs clinicians (TU and DP) who were site principal investigators on the trial and provided respondent validation [20]. The two researchers (HL and LM) coded the remaining interviews equally, drawing up memos for each interview to provide additional context for other researchers analysing the data, and recoded the original twelve interviews. Minor, iterative changes to code definitions were made. An audit trail was kept.

To address the aims of this study, provider and patient experiences that were related to trial implementation which were coded to: ‘trial impact’ and ‘research motivation’ and ‘real world’, were explored.

Additionally, some triangulation of findings was obtained through a review of existing Kanyini GAP documents (e.g. Email communications, Memoranda of Understanding, and financial statements) [17].

The study was approved by 7 regional committees including 1 Aboriginal-specific committee jurisdiction (Sydney South West Area Health Service (HREC Ref. 08/RPAH/126); Aboriginal Health and Medical Research Council of NSW (642/08); Cairns Base Hospital (HREC/08/QCH/10-546); Princess Alexandra Hospital Centres for Health Research (HREC/08/QPAH/160); Central Australian Human Research Ethics Committee (2008.09.04); Northern Territory Department of Health and Menzies School of Health Research (HREC 2010–1466; Monash University Human Research Ethics Committee (CF09/2353 – 2009001370)).

## Results

At the end of Kanyini GAP, 94 semi-structured interviews were conducted by the interview team with 47 providers (25 GPs, 13 pharmacists, 6 Aboriginal health workers (AHW) and 3 chronic care nurses) and 47 patients in NSW, Queensland and Victoria. There were 22 and 25 patients who were in the polypill arm and usual care arm, respectively. There were 26 non-Indigenous patients and 21 Indigenous patients.

The critical enablers and barriers to implementing this PRCT in Australian primary healthcare settings were broadly grouped within three key themes: recruitment and participation; research and primary practice settings; and participant costs. Tables 1 and 2 summarise the identified barriers and enablers to conducting a primary care PRCT within these three key categories and presents suggested strategies to overcome barriers and maximise enablers when implementing future PRCTs in primary practice settings.

**Table 1:** Strategies to overcome barriers to implementing primary care pragmatic randomised controlled trials

Barriers	Strategies to help overcome
Recruitment and participation	
Prescriber disagreement about recruitment across sites: clinical eligibility compared to trial suitability	• Prior to implementation, identify potential sources for disagreement, provide examples and workshop solutions with providers
	• Throughout recruitment, facilitate a forum for providers to discuss with research team actual difficulties encountered
Potential negative impact of evaluated intervention on provider’s business revenue	• Identify and discuss potential impacts (immediate and long-term) with providers;
	• If possible, ensure lost revenue adequately compensated
	• Educate potential providers about the value of the intervention to public good
Highly mobile patients	• Consider provision of mobile recruitment services
Research and primary practice settings	
Inadequate research infrastructure	• Ensure adequate physical space available for trial processes
	• Understand information technology (IT) capacity at sites and use study systems that can integrate with pre-existing IT, thus minimising training requirements
	• Consider using data extraction tools to minimise access time to information technology systems
	• Ensure adequate remuneration to participants for time and service provided
	• Consider provision of dedicated research coordinator at sites, particularly those already understaffed
Pre-existing workforce strains	• Adequately understand workforce-related issues at participating sites
	• Ensure adequate personnel support is available and can respond to high staff turnover
	• Ensure adequate training at practice level, and refresher training available and budgeted for
Potential miscommunication across multidisciplinary health services beyond primary care	• Provision of simple communication tools at the patient and practice levels that highlight patient involvement within the trial.
	• Adequately educating patients and carers regarding about trial and need for communicating to all healthcare providers
	• Identify participant multidisciplinary providers at enrolment and target trial communication strategies accordingly
Increased administrative burden relative to health service delivery and patient care demands	• Provide adequate research support to sites that can minimise administrative burden
	• Consider automated procedures that ensure Good Clinical Practice compliance and can integrate with current health service processes
	• Ensure site service delivery requirements are fully understood prior to implementatin
	• Provide clear education about Good Clinical Practice
	• Practice requirements and administrative needs prior to recruitment
Costs	
High trial running costs	• Ensure adequate budget for provision of research support personnel at sites to maintain recruitment timelines, ease administrative burden to sites and reduce opportunity cost to sites
	• Additional funding load to accommodate inadequate primary care research infrastructure
	• In medium to long term establish a funding pool to invest in primary healthcare research infrastructure
Opportunity cost to participants	• Understand potential costs to participants prior to implementation
	• Provide adequate remuneration to participants in light of actual time required for administration, including time spent with research nurses
	• Ensure simple processes for sites to apply for and receive remuneration

**Table 2:** Strategies to maximise enablers to implementing primary care pragmatic randomised controlled trials

Enablers	Strategies to help maximise
Recruitment and participation	
Leveraging pre-existing networks and relationships with key stakeholders	• Provide adequate pre-recruitment engagement with stakeholders and elicit expressions of interest
	• Engage with stakeholders at trial-design stage to build a sense of ownership and address research objectives of participants
Increased research capacity	• Understand research needs of sites and fulfil gaps in research capacity as requested
	• Incorporate capacity building as a key outcome for participation
	• Provide opportunity for training at health service level to build research capacity within primary healthcare.
Research as a quality service indicator and team building exercise	• Provide structured training for sites as a means for team building between and across sites
	• Research participation as a quality assurance indicator for primary practices: policy development consideration
Professional support for the intervention under evaluation and tangible benefits to the service or participant	• Understand and address professional concerns about the intervention under evaluation
	• Promote the potential benefits of trial participation to health service and participants
Personal and community benefits research participation	• Understand and promote benefits (and risks) of research to individuals and community
	• Educate participants about research goals and needs
	• Ensure participants feel sufficiently empowered to make decisions about ongoing participation
Research and primary practice settings	
Provision of research coordinator	• Prior to implementation, proactively identify site resource needs in terms of trial-related administration, communication, data management and patient management
	• Ensure adequate research and logistical support is provided

### Recruitment and participation

#### Site recruitment

The recruitment process for enlisting sites included a number of initial meetings, workshops and dinners to introduce the trial and elicit expressions of interest. This approach proved an effective way to develop and strengthen existing relationships with primary care services. Kanyini GAP also built upon previous research done with the IHSs, which found a gap in the prescribing of indicated CVD medications; thus site recruitment to Kanyini GAP was seen as a ‘natural progression’ (Provider 47, AHW). This is evident from the following comment from a GP based at an urban IHS:

‘*The other thing that was really helpful was the way the service was engaged by The George Institute so that the community all knew about polypill. They’d engaged with the Board very well, the Board and the service had agreement so the CEO and the manager in the service knew about it. They’d held a launch day at the service and people were asking questions, so there was a lot of engagement, a lot of patients knew about it, so there was general awareness.*’ (Provider 8, GP)

Some participating GPs envisaged tangible benefits to their practice through involvement in primary healthcare research, in terms of quality improvement and staff morale. As a GP at an urban private practice site describes:

‘*Philosophically I like the idea as a practice being opened up to researchers …… I’ve done a lot of quality improvement with practices and one of the things that builds the team is opening it to the outside world. And, participating in research is a way of opening it to the outside world, so it’s actually a plus for team building. Practices feel proud that they’re actually working at this level.*’ (Provider 1, GP)

In addition to staff morale, a collective increase in research capacity at the health service level was thought to be a positive impact of participating in primary care research, as one GP from an urban IHS noted:

*‘It was good for our research capacity, it was a project we all believed in and got behind. … our name came up just on the weekend at the conference that I was at … people mentioned that we were part of the Kanyini GAP trial. So I think from that point of view it was good for our health service, good for our reputation. … participating in research I think was a good experience for those workers some of whom were Aboriginal, so that’s increasing the research capacity, Indigenous research capacity for (the) clinic which is a good thing.*’ (Provider 33, GP)

#### Providers’ research motivation

Motivation to take part in the research study was frequently mentioned, with many providers stating that being part of a trial is for the ‘greater good’. The following GP from an urban IHS outlined her motivation to take part in investigator-led research:

‘*I really liked and felt comfortable and trusted the Kanyini polypill … that it was put together on the basis of what was going to be best for people with cardiovascular disease rather than the profit motive which pharmaceutical companies have to go by because they’re private companies and have shareholders.*’ (Provider 33, GP)

Many of the pharmacists participated because they were interested in the intervention under evaluation. Several were candid about the potential negative effect a polypill strategy could have on their revenue. As described by a pharmacist in an urban site based close to an IHS:

‘*Now I’m aware of different critical remarks among community pharmacists … instead of three or four dispensing fees we see one … not being a pharmacy owner … I’m less sensitive to that issue … By and large it’s good for customers, it’s good for compliance, it’s good for the government I suppose.*’ (Provider 15, Pharmacist)

For some, participating in a trial was also thought to create an opportunity for health professionals and services to improve the care given to patients while promoting collaborations with other organisations, as this next Aboriginal health worker from a remote IHS described:,

‘*I’d say well it gives them (the health service) the opportunity to give their patients the best possible care that they can … offer this one more thing (that) can actually influence a lot of the patients that we have. So that offers better care for our patients. But then, you know, helping out studies also yields partnerships with people, other organisations.*’ (Provider 41, AHW)

#### Recruitment of patients

Patient recruitment in the pragmatic trial required substantial effort due to the diversity of the health services and broad characteristics of patients expected to participate. For instance, recruitment in remote services was particularly challenging given patient populations were highly mobile and health considerations complex. A GP in a remote IHS described it as such:

‘*I think there's a difference between being eligible and suitable, so I think that was something that wasn't really teased out properly. We got, I think, nearly close to our target, because we recruited a lot of eligible patients. In terms of suitability, I don't know whether we really picked patients that were appropriate for a trial.*’ (Provider 40, GP)

Moreover, for this next GP, describing the recruitment of patients with complex comorbidities from private practice, the broad patient eligibility criteria proved a source for disagreement between prescribers about recruitment:

*‘I was surprised that some of the patients, who others had been happy to put on it because when we looked at the problems that some had, I thought, well I wouldn’t have put that person in, in the first place… Because they hadquite acomplicated history and potential risks of having some problems … I wanted them to either fit in clearly or not … So I suppose I was looking for people who didn’t really have lots of other comorbidities … I would have said, “They’re not suitable”.’* (Provider 36, GP)

#### Patients’ research motivation

For patients, an overwhelmingly positive response to being involved in the trial was expressed with many claiming they were happy to be involved as ‘guinea pigs’ and to play a part in contributing to ‘finding a cure’. Taking part in the trial was thought to not only offer potential benefit to them as individuals, but to others as well, and Aboriginal and Torres Strait Islander people in particular expressed their interest in the trial for this reason:

‘*You know there’s something you’re contributing to, and it’s not just about you; it’s about how it might help the rank and file right across the nation … if I can help my people live longer, live better lifestyles, healthier lifestyles, then I want to be a part of that. I just want to be part of that group that does that.*’ (Patient 10, urban IHS)

This ‘big picture’ thinking translated to a willingness to be involved, to trust in the health system and a sense of doing something meaningful for others even if there were no evident or immediate benefits for themselves:

‘*I don’t have a problem with studies. I think if it’s going to ultimately benefit mankind, I'm happy to sort of be a bit of a guinea pig. It’s an interest. It’s also a possibility that I will suffer better health because of it, so I don’t have a problem with those types of things generally.’* (Patient 16, of private GP)

**‘***Well I did feel an obligation not to withdraw and I don’t know how many people in your control group stuck it out to the end but you need a certain number to you know, validate the statistics … But I never felt any pressure, I was always assured that I could pull out at any time if I’d had enough. But the pressure came from within, you know… I’ve started I should finish.*’ (Patient 25, of private GP)

### Research and primary practice settings

#### Research infrastructure

Overwhelmingly, providers cited inadequate infrastructure as a substantial barrier to trial implementation. Infrastructure considerations included: physical space to conduct patient visits, access to information technology systems, and storage space for additional supplies of polypill (for post-trial provision of polypill to participants). Time, money and human capacity were other necessary resources which were reported as being limited. A pharmacist in a remote area described such challenges:

‘*… I guess from our perspective, and it came down to not necessarily The George Institute, but it was more our settings and our dispensing program and also staff education as well …*’ (Provider 42, Pharmacist)

#### Research and logistical support

The provision of a research coordinator at the study sites was described by many providers to be a key facilitator to trial implementation. In IHSs, the research nurse provided logistical support for the trial through trial-related communication with health service staff members, administration, obtaining informed consent, and data collection. This is evident in the following comment from a GP at an urban IHS:

‘*I think it has been a good thing. It's not an added, the admin, the workload doesn’t add on because we have the team for support here. So in that way it wasn’t even, didn't even notice. And it's just like any other pill really, just prescribe it. It was easy enough; it was already on our system so we just prescribe it just like any other.*’ (Provider 5, GP)

A GP and medical director in an urban private practice also thought that the research nurse facilitated the conduct of the study and, therefore, alleviated the effort required from the GPs in her service:

‘*… she (research nurse) facilitated everything brilliantly. … we cringe sometimes when people ask us to do studies in a busy general practice … without the nurse it would have been a nightmare really, … Well, I think the whole study would not have worked without her, and I think it’s a real lesson for any GP research is having a research nurse is key.*’ (Provider 12, GP and Medical Director)

#### Workforce-related issues

Some sites with particularly large patient loads, long clinic waiting times and many rotating GPs faced challenges due to potential for miscommunication between providers, and a lack of general understanding of the study across the service. Integrating the intervention within the realities of a dynamic workforce and chronic staff shortages was particularly difficult. For instance, at some IHSs, the research coordinator position proved to be difficult to replace as required, due to a complicated trial coordination handover and the need for staff re-training. A medical director of an IHS describes the effect of high staff turnover on trial conduct:

*‘I think it was more good fortune than anything else that we actually made it to the finishing line to tell you the truth … staffing’s been a problem the whole way through really. I think we’ve actually had about three or four sort of individuals that have been identified as actually the local supports or go-to people for the trial … within a period of 18 months, 2 years.*’ (Provider 46, Medical Director)

It was also acknowledged that this problem was likely compounded by the ongoing problem of workforce retention in remote settings.

#### Administrative demands of clinical trials

The paperwork requiring compliance with Good Clinical Practice guidelines, including the reporting of adverse events, was highlighted by some GPs as being an unacceptable additional workload. As one GP in private practice stated:

‘“*It’s the paperwork (that) has driven me crazy … I don’t see any future for research if that’s the amount of paperwork you’ve got to see, … I can understand why you’ve got to do it but it’s just insane and I think people who design these things and make the rules ought to go and have a good hard look at themselves and say you know, this is stupid … Now you know, adverse reactions are important but you know, most of them are rubbish, most of them have got absolutely nothing to do with the study.*’ (Provider 10, GP)

When asked about future involvement in a PRCT, this GP in an urban setting was negative about his experience, questioning the feasibility of doing ‘research on people in the “real world” … properly’, (Provider 10, GP). This scepticism was based on the trial’s administrative demands competing with his fundamental priority of adequate service delivery and patient care.

In contrast, some providers did not find the additional paperwork overly burdensome, acknowledging time was required to be spent on training and administrative paperwork as a condition of committing to trial participation.

#### Multidisciplinary care beyond primary care

Another area of difficulty identified with implementing a trial in primary care settings was that it involves patients who require multidisciplinary care that may be received outside of the primary care sites involved in the trial. In particular, difficulties with communicating information about the trial between primary and secondary healthcare was described to potentially impact patient retention, as this next GP stated:

‘*… a person that goes in and out of hospital. In which case I think it just caused a stress. Because they’d go in and then they’d have all of the interns and then the residents and registrars and everybody … What is this thing and what are you on, … And how do we, and what do we do and how we got to change this? So I had a couple of times when patients would actually come and just felt that it was too difficult because of their multidisciplinary care.*’ (Provider 3, GP)

Although extensive efforts were made to inform stakeholders about Kanyini GAP at the outset (e.g. informing specialists about the study prior to commencement, explaining the polypill within referral letters and providing information cards to patients), and these were described by providers as an essential ‘safeguard’ against miscommunication, the above finding suggests that such measures may not have been sufficient.

### Participant costs

Participants did not indicate that the incentive payments provided for participating in the Kanyini GAP trial influenced their involvement in the study, suggesting that the altruistic motivations outlined earlier were primarily considered. Furthermore, not all pharmacies claimed their entitlement offered to support the dispensing and handling of the polypill. Some pharmacists reported the ‘small’ payments were not worth the time and effort involved in preparing the necessary paperwork.

Despite our measures to minimise the financial impact through a remuneration of AUD$100 per patient randomised into the trial, a number of GPs from private clinics did highlight that the time involved in taking part in the trial carried a significant opportunity cost. The following comment from a GP, who was a private practice proprietor located outside an urban area, indicates that even with the provision of a research nurse, participation resulted in an opportunity cost:

‘*You run a business, you really can’t, (take that much time out), and even so we still spent quite a bit of time with (the research nurse)… I don’t know how many appointments we missed because of time with her even though it wasn’t huge, it adds up.*’ (Provider 37, GP and private practice owner)

## Discussion

### Summary

By exploring provider and patient experiences from Kanyini GAP, and relevant trial materials, our analysis has revealed considerable barriers to implementing a PRCT in primary care. Specifically, a substantive lack of research infrastructure, limited numbers of primary care personnel adequately trained in the conduct of clinical trials, administrative burden from regulatory requirements that exceeded the demands of adequate patient care provision and the lack of coordination across all providers involved in the treatment of patients, including non-primary healthcare providers, substantially impeded implementation. Additionally, the ongoing problem of an under-resourced primary care workforce meant that centrally employed research nurses were needed to support the sites. As a consequence of these barriers, funding for this study – around AUD$5 million and sourced from multiple sponsors – ultimately proved insufficient. As a result, recruitment timelines were longer than anticipated and ultimately targets were not met.

Despite these shortcomings, participating in Kanyini GAP was generally considered a positive experience with mutual benefits stated for patients and providers involved. Benefits included professional satisfaction, increased collaboration between the different health services involved, improved research capacity and the opportunity for health services to improve patient care. In addition, patients and providers participated for altruistic reasons, being particularly motivated by the chance to contribute to the ‘greater good’. The success of completing Kanyini GAP appears largely attributable to an upfront investment to build and maintain collaborations across the diverse range of Australian primary healthcare settings and, notwithstanding the additional financial cost incurred, from an intensive level of research support provided to participating sites.

### Recruitment challenges in PRCT

The challenges of meeting recruitment targets particularly within PRCTs have been well-documented [9, 22–24]. A meta-analysis of interventions to promote patient recruitment to primary care concluded that organisational characteristics, especially trial infrastructure, were important [23]. Similarly, our findings indicate that a lack of such research infrastructure in Australian primary practice contributed substantially to recruitment delays. However, the high level of research motivation reported from both providers and patients that was underpinned by a sense of altruism facilitated recruitment.

Some design features of Kanyini GAP, classified with the PRECIS tool as more pragmatic than explanatory, presented further challenges to recruitment and implementation. Specifically, the ‘participant eligibility criteria’ domain was highly flexible in Kanyini GAP such that all patients with the condition of interest were considered eligible [10, 11]. Using these criteria to assess site feasibility on the basis of predicted recruitment targets led to an overestimation of participant numbers compared to what could be achieved in practice. Furthermore, the domain ‘experimental intervention practitioner expertise’ allowed for a full range of practitioners to apply the intervention within the clinics [10, 11]. In larger sites that had multiple staff on rotation, more training and logistical support for practitioners was required throughout the time frame of the study than might have occurred if selected personnel were responsible for applying the intervention. Collectively, these pragmatic design features of Kanyini GAP meant there was a need to engage more sites, extend study timelines and increase expenditure to try and meet recruitment targets. However, the use of these pragmatic criteria is necessary as they allow for real practice variation and an assessment of the acceptability and generalisability of the intervention.

### Challenges unique to trial implementation in a primary care setting

Our study has identified some additional challenges which may be unique to the conduct of a clinical trial in the primary care setting. First, in contrast to research traditionally conducted in public healthcare facilities, a number of primary healthcare providers in Kanyini GAP were operating in the private sector. Although altruistically motivated to participate, the impact of the trial on revenue and time was an important consideration. Furthermore, despite establishing PRCT research partnerships that would now be classified as ‘best practice’, (e.g. site feasibility pre-assessment, stakeholder involvement and integration into usual practice workflow) [25], these efforts were insufficient to mitigate the burden that was experienced by some Kanyini GAP providers. In this regard, identifying and discussing the immediate and long-term financial impact of the trial with the healthcare providers is important at the outset. Compensation for such costs needs to be built into existing funding models for pragmatic trial research.

Second, as most primary care sites in Kanyini GAP were independently owned, there was substantial variation in the day-to-day operation between sites. Chronic staff shortages and high staff turnover were problematic, particularly at rural and remote sites where some of the most disadvantaged and difficult-to-reach patients reside. To enable the streamlined integration of the trial with usual primary care processes, the provision of intensive research support is needed via trained research nurses. Research conducted in greenfield sites, involves significant investment to increase their research capacity for future studies.

### Strengths and limitations

This study has identified enablers and barriers to the conduct of a PRCT in Australian primary healthcare settings by directly considering the experiences of participants. This information is vital for clinical researchers who seek to generate ‘real-world’ evidence to bridge the significant gaps known to exist between the controlled trial environment and practice. However, this study did not include the remote Central Australian sites involved in Kanyini GAP. Thus, the generalisability of the study’s findings to such sites, and to other healthcare systems cannot be certain. So as not to influence the adherence behaviours of participants, interviews could not be conducted until the end of study. Invariably, the opinions of participants who had dropped out of the trial prior to the end-of-study visit, or sites that were approached but declined to participate, could not be ascertained. However, the rigorous methods used in this study, particularly triangulation of data sources, using more than one interviewer and coder, and the breadth of clinical and research experience of the research team favour robust results.

## Conclusions

A number of key recommendations for the implementation of future PRCTs in primary care have emerged. First, significant investment in primary care research infrastructure is needed to facilitate recruitment and successful trial completion. Information technology systems that streamline data capture relating to key outcomes (e.g. hospitalisation and mortality) and that can promote communication across the various health system levels (e.g. primary and secondary care) is one suggestion.

Second, building research capacity within primary care is essential. Including research as a key performance or quality assurance indicator may increase research capacity, albeit indirectly. The increased exposure of patients and practitioners to research may ultimately lead to PRCTs being viewed as a standard feature of high-quality primary healthcare services. This is congruent with experiences of other PRCT trialists which found that conducting PRCTs has the potential to achieve greater partnerships between researchers and healthcare systems to produce high-quality studies to improve health-care [25].

A final strategic recommendation would be sustained funding for adequately resourced primary care research networks, incorporating private practices, ACCHSs and pharmacists. Based on international evidence [26], practice-based research networks (PBRN) are now starting to emerge in Australia but are currently poorly funded [27].

Notwithstanding the development of such networks, sufficient resourcing must be set aside for individual projects to cover the full costs of involving large numbers of disparate stakeholders in research. It is important to recognise that the high unbudgeted costs in the Kanyini GAP trial were to accommodate the lack of research experience and training in primary care. Substantial costs associated with running trials in primary care settings are incurred upfront, particularly when partnering with numerous centres which have had limited or no research experience. Such upfront costs should include not only costs to the study but the burden to the individual centres for which, as uncovered in our interviews, was often uncompensated for.

By initiating research across a numerous set of diverse sites, Kanyini GAP has cleared paths for easier and less costly implementation of future Australian primary care PRCTs. A key recommendation from this project, therefore, is that recognition of such path-clearing investments is required and that provision either be made (in the short term) for loadings on research funding for new projects that specifically set out to perform similar roles or (in the medium to long term) the creation of a general investment pool to fund primary care research infrastructure. Such initiatives will encourage investment in capacity that will contribute to a broader research environment more conducive in the long run to the running of much-needed PRCTs.

## Additional files

Additional file 1.
**Populated CONSORT flow diagram of the completed RCT.** (DOC 47 kb) Additional file 2.
**CONSORT checklist of the completed RCT.** (DOC 217 kb) Additional file 3.
**Coding framework.** (DOCX 21 kb)

## Abbreviations

ACCHS: Aboriginal Community Controlled Health Service
AHW: Aboriginal health worker
CONSORT: Consolidated Standards of Reporting Trials
CVD: cardiovascular disease
GP: general practitioner
IHS: Indigenous health service
Kanyini GAP: Kanyini Guidelines Adherence to Polypill Study
PBRN: practice-based research networks
PRCT: pragmatic randomised controlled trial
PRECIS: Pragmatic-Explanatory Continuum Indicator Summary
RCT: randomised controlled trial
