# Supplementary material for: Implementing Kanyini GAP, a pragmatic randomised controlled trial in Australia: findings from a qualitative study
Source: Trials. 2015 Sep 23;16:425. doi: 10.1186/s13063-015-0956-y (PMC4581084; doi:10.1186/s13063-015-0956-y)
Supplement: Additional file 1. — Populated CONSORT flow diagram of the completed RCT. (DOC 47 kb) [file 13063_2015_956_MOESM1_ESM.doc]

**
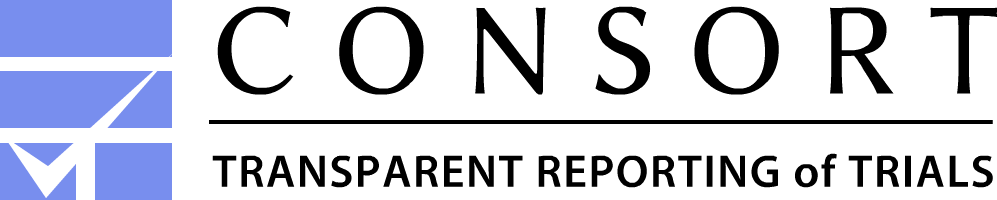
**

**APPENDIX A: CONSORT 2010 Flow Diagram for Kanyini GAP**

**Allocation**

**Analysis**

**Follow-Up- visit at 1 Month +6, 12 18, 24, 30 and 36 months**

**Enrollment**

Assessed for eligibility (n=731)

Excluded (n= 108 )

♦  Not meeting inclusion criteria (n=108)

Analysed (n=311)

Lost to follow-up (Patients Dead=1, Refuses further participation=3, unable to contact=3) (n= 7 )

Discontinued intervention (Patients’ choice, discontinued by treating doctor, discontinued while hospitalised, Side effects) (n=84)

Allocated to polypill-based strategy (n=311)

BP measures =308

Total Cholesterol measures=310

Lost to follow-up (Patients dead=1, Refuses further participation=3, unable to contact=2, Missing n=1) (n=7)

Discontinued intervention N/A

Allocated to usual care N=312

BP measures=309

Total Cholesterol measures=310

Analysed (n= 312)

Randomized (n=623)
