# Supplementary material for: Implementing Kanyini GAP, a pragmatic randomised controlled trial in Australia: findings from a qualitative study
Source: Trials. 2015 Sep 23;16:425. doi: 10.1186/s13063-015-0956-y (PMC4581084; doi:10.1186/s13063-015-0956-y)
Supplement: Additional file 3. — Coding framework. (DOCX 21 kb) [file 13063_2015_956_MOESM3_ESM.docx]

**FINANCIAL CONSIDERATIONS**Closing the Gap policy, Pharmaceutical benefit scheme, Safety Net, Cost of medications & health care

Additional File 3: Coding Framework

**ABORIGINAL HEALTH CONSIDERATIONS**
- social justice
- determination
- cultural safety

**REAL WORLD**

**- Population Health Approach**
- use of absolute risk
- primary prevention
- ‘ideal patient’

**– ‘On the shelf’**- policy implications
- future polypill combinations

**TRIAL IMPACT**
- current treatment
- patient management
- effects on services, (e.g. bottom line, time)
- effects on patient, (e.g. changes in med-taking behavior, increased health awareness)
- tailoring of meds

**RESEARCH MOTIVATION**
- greater good
- personal good

**BEING WELL**
- self care
- general health
- family and community support
- stress

**GOOD CARE**
- provider-patient relationship
- access to health services
- other support services

**HEALTH LITERACY**
- health-seeking behavior
- missed dose effect

**ACCEPTABILITY OF POLYPILL**
- advantages: less tabs, ease, convenience, cost
- disadvantages: fixed combination, side effects, tailoring of meds

**ADHERENCE**- strategies for adherence
- complexity
- burden of pills
- drug holiday

-

**PEAK Coding Framework**
